# Atypical progression of Group B Streptococcus infection: Subdural empyema in an adult with diabetes mellitus

**DOI:** 10.1016/j.idcr.2025.e02212

**Published:** 2025-03-31

**Authors:** Jo Onaka, Takahiro Fukushima, Akihito Yoshida, Nicole Leedy, Takaaki Kobayashi, Kyoichi Tomoto, Kazuaki Aoki

**Affiliations:** a Department of General Medicine, Kameda Medical Center, Kamogawa, Chiba, Japan; b Department of Internal Medicine, Division of Infectious Diseases, University of Kentucky, Lexington, KY, USA; c Department of Neurosurgery, Kameda Medical Center, Kamogawa, Chiba, Japan

**Keywords:** Group B Streptococcus, *Streptococcus agalactiae*, Meningitis, Subdural abscess

## Abstract

*Streptococcus agalactiae* (Group B Streptococcus, GBS), traditionally associated with neonatal meningitis and urinary tract infections in pregnant women, has emerged as a significant pathogen in non-pregnant adults. A broad spectrum of GBS infections in adults has been reported, including skin and soft tissue infections, bacteremia without a clear source, urinary tract infections, pneumonia, and less commonly, endocarditis, meningitis, or other invasive infections. We report a rare case of subdural empyema following GBS bacteremia in a 74-year-old man with poorly controlled type 2 diabetes mellitus. The patient presented to the outpatient clinic with progressive gait instability persisting for five days, preceded by a resolved headache and diarrhea. On examination, he was febrile but exhibited no nuchal rigidity or focal neurological deficits. He was discharged home, but blood cultures subsequently grew *S. agalactiae*, prompting emergent hospital admission. Initial neuroimaging, including magnetic resonance imaging (MRI) of the brain, was unremarkable. On hospital day 5, the patient developed worsening altered mental status and right upper limb weakness. A lumbar puncture confirmed GBS meningitis and repeat brain MRI revealed a subdural empyema. The patient underwent surgical drainage and received prolonged antibiotic therapy, resulting in significant clinical improvement. This case underscores the importance of maintaining a high index of suspicion for meningitis and subdural empyema in patients with GBS bacteremia who develop new neurological symptoms, even when initial imaging is unremarkable. Early recognition, repeat neuroimaging, and timely intervention are essential for managing invasive GBS infections and improving patient outcomes.

## Background

*Streptococcus agalactiae* (Group B Streptococcus, GBS) is the leading pathogen responsible for meningitis and sepsis in neonates [1]. Although GBS is an uncommon cause of bacterial meningitis in adults, it can lead to severe central nervous system (CNS) infections, including brain abscess [2]. The disease may exhibit rapid progression and subdural empyema is atypical to GBS in adults, posing diagnostic challenges for clinicians. GBS in adults commonly causes bacteremia without a focus, skin and soft tissue infections, endocarditis, osteomyelitis, pneumonia, and urinary tract infections, with varying presentations and severity depending on the site of infection and the patient's underlying health conditions. [3] In this case report, we describe the clinical course of a 74-year-old man with diabetes mellitus who developed subdural empyema following GBS bacteremia, despite normal initial magnetic resonance imaging (MRI) findings.

## Case

**Fig. 1:**
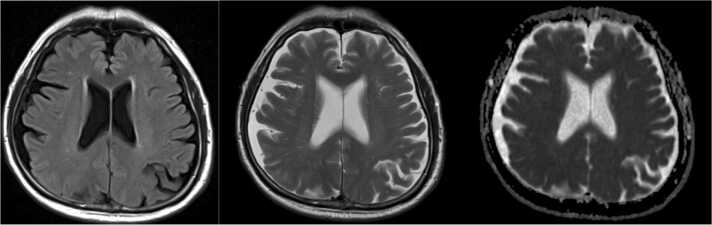
The initial MRI on presentation had no abnormal findings.

A 74-year-old man with a history of type 2 diabetes mellitus presented to the outpatient clinic with progressive gait instability persisting over five days. Notably, he had experienced headache and diarrhea three days prior, which had resolved one day before this visit. He denied nausea or vomiting. His social history included previous smoking and moderate alcohol use, consuming two alcoholic beverages per week. On presentation, he was febrile, with other vital signs within normal limits. Neurological examination showed no nuchal rigidity or focal neurological deficits. The remainder of his physical exam was unremarkable. Initial laboratory tests revealed a peripheral leukocytosis with a white blood cell count of 17.3 K/uL (reference range 3.3–8.6 K/uL) and neutrophilia. Given a suspicion of an intracranial lesion, brain MRI was performed, revealing no abnormalities (Fig. 1). Computed tomography (CT) of the neck, chest, abdomen, and pelvis, performed to evaluate for potential abscess formation, also revealed no findings suggestive of infection. Considering the elevated inflammatory markers and his diabetic status, blood cultures were obtained while antibiotic therapy was not initiated. He was discharged home with plans for close follow-up.

**Fig. 2:**
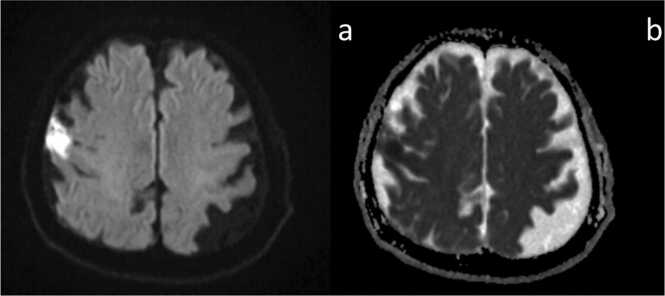
The repeat MRI (a, b: DWI) on the second day of admission. The diffusion-weighted image (a) demonstrates an area of marked high signal intensity in the subarachnoid space lateral to the right frontal lobe, accompanied by a slight decrease in the ADC value (b).

Unfortunately, the blood culture became positive after 12 h of incubation, with Gram-positive cocci detected. Matrix-Assisted Laser Desorption/Ionization Time-of-Flight Mass Spectrometry (MALDI-TOF MS) identified the isolate as *Streptococcus agalactiae*. A urine culture obtained at presentation was negative for bacteria. He was contacted by phone and instructed to return to the hospital for admission. On arrival, he had a temperature of 36.4°C, heart rate of 100 beats/minute, respiratory rate of 18 breaths/minute, and blood pressure of 118/99 mm Hg. His physical exam was notable for spinal tenderness in the lumbar region. Repeat laboratory workup indicated a white blood cell count of 9.6 K/uL with neutrophilia and a C-reactive protein (CRP) level of 316.6 mg/L (reference range 0–1.4 mg/L). His kidney function was within normal range (Creatinine 0.72 mg/dL, BUN 14 mg/dL). His liver function test was found to be slightly elevated: alanine aminotransferase (ALT) 52 U/l (reference range 0–33), aspartate aminotransferase (AST) 26 U/l (reference range 0–32) and gamma-glutamyl transferase (GGT) 81 U/l (reference range 0–40). Considering the preceding diarrheal symptoms and the possibility of the intestine as an entry portal for infection, ampicillin-sulbactam was administered for coverage against anaerobic bacteria as well as his known GBS bacteremia. On the following day, two sets of admission blood cultures were also positive for *S. agalactiae* after 13 h of incubation. *S. agalactiae* isolated from the blood was susceptible to penicillin G (Table 1); therefore, the antibiotic regimen was narrowed to penicillin G. On the third day of admission, lumbar spine MRI revealed L4–5 intervertebral discitis. That same day, the patient exhibited a decline in pulse oximetry and a mild reduction in consciousness. However, due to the absence of significant neurological findings with the initial negative brain image, intracranial pathology was considered unlikely, and the same treatment was continued without repeat imaging. On the fourth day, the patient experienced worsening altered mental status and right upper limb weakness, prompting re-evaluation with brain MRI. Diffusion-weighted imaging (DWI) revealed hyperintensity in the cerebrospinal fluid surrounding the frontal lobe, raising suspicion for meningitis and subdural empyema (Fig. 2).

**Table 1:** Antibiotic susceptibilities of *S. agalactiae* isolated in blood cultures.

**Antibiotic**	**MIC interpretation**
Penicillin G	S
Erythromycin	S
Clindamycin	S

**Fig. 3:**
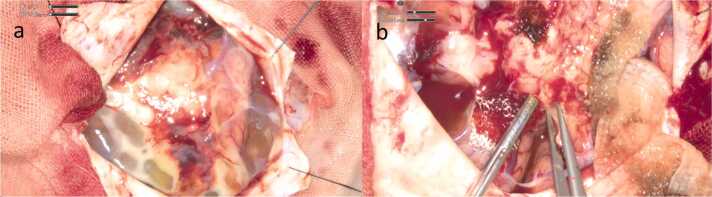
Operative findings of the surgical abscess drainage. In Fig. 3a, the abscess was strongly adherent to the pia mater, with an unclear boundary between the abscess and brain parenchyma. In Fig. 3b, the abscess was removed, along with the disrupted pia matter and a portion of the cortex, while preserving the motor cortex.

On the fifth day, a lumbar puncture was performed. Cerebrospinal fluid (CSF) appeared slightly yellow with xanthochromia, revealing a cell count of 140/µL, protein concentration of 325 mg/dL, and glucose level of 76 mg/dL. Although CSF culture was ultimately negative, FilmArray testing detected *S. agalactiae*. On the same day, the patient experienced tonic-clonic seizures with conjugate gaze deviation, prompting initiation of a prophylactic antiepileptic regimen with levetiracetam. Although transthoracic echocardiography (TTE) was normal, a splinter hemorrhage was noted on the fourth finger of the right hand, raising suspicion for infective endocarditis. Consequently, gentamicin was added to the treatment regimen on the fifth day and continued for two weeks with the aim of achieving a synergistic effect with penicillin. A neurosurgery consultation was obtained for subdural empyema, and abscess drainage surgery was performed the same day. An abscess adherent to the pia mater and partially invading the brain parenchyma overlying the motor cortex was identified (Fig. 3) at the time of surgical evacuation. The abscess was excised as completely as possible, sparing areas indistinguishable from the brain parenchyma. Cultures from the abscess grew *S. agalactiae*, sensitive to penicillin.

**Fig. 4:**
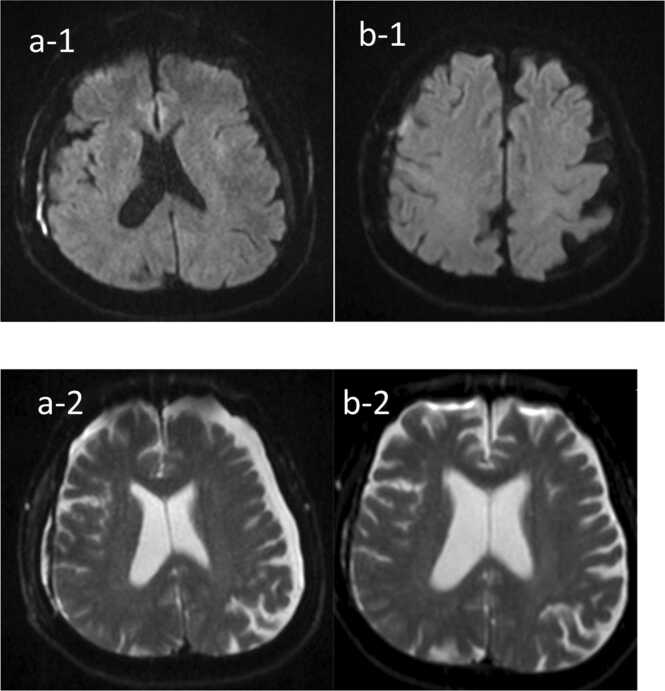
The brain MRI on 31th (a-1, a-2) day and 2weeks after discharge (b-1, b-2). [a-1], [a-2] The abscess on the surface of the right frontal lobe shows signs of shrinking. However, a new abscess has appeared in the right lateral subdural space of the frontal lobe. [b-1], [b-2] The abscess on the surface of the right frontal lobe shows further shrinkage. The abscess in the right lateral subdural space of the frontal lobe, newly observed in the previous MRI ([a-1], [a-2]), has also decreased in size.

Following surgical drainage, there was marked improvement in both inflammatory markers and clinical status. Since the initiation of levetiracetam, the patient experienced no further seizures. On day 31, follow-up MRI revealed findings initially suggestive of a new abscess. However, after consultation with the radiology team, these findings were attributed to the spread of residual abscess rather than the formation of a new lesion (Fig. 4-a). Consequently, the existing antimicrobial regimen of IV penicillin G was continued. The patient completed an 8-week course of IV penicillin and was discharged in stable condition. At the two-week follow-up, he remained asymptomatic with no signs of active infection. Repeat MRI demonstrated a reduction in the size of the subdural empyema and no evidence of new lesions (Fig. 4-b).

## Discussion

GBS is an encapsulated, β-hemolytic, Gram-positive coccus commonly colonizing the gastrointestinal tract, respiratory system, and vagina. Although GBS is the leading cause of meningitis and sepsis in neonates, it can present with diverse clinical manifestations in adults, including bacteremia, meningitis, and endocarditis [3]. While GBS accounts for roughly 1 % of adult meningitis cases [4], a recent rise in GBS meningitis among adults suggests a potential shift in the disease spectrum associated with GBS [5].

Risk factors for GBS meningitis in adults include conditions such as diabetes mellitus, cardiac disease, pregnancy, postpartum status, extra-meningeal infection foci, and CSF leakage [6]. Diabetic individuals, in particular, have a roughly 10-fold increased risk compared to healthy adults [7]. However, there are cases of adult-onset GBS meningitis occurring in otherwise healthy adults without apparent risk factors [8]. Although our patient had diabetes, it was unclear whether meningitis was the source or a consequence of bacteremia. The CSF findings in adult GBS meningitis closely mirror those of other bacterial meningitis types. However, GBS meningitis carries a significant risk, with a reported mortality rate of 27–34 %—notably higher than that seen in meningococcal meningitis [9]. Endocarditis is the most common extra-CNS infection focus associated with GBS meningitis, and spondylodiscitis has been reported as a complication in some cases [6]. In our patient, MRI findings two days post-admission suggested spondylodiscitis, though the primary infection source was unclear. Gastrointestinal translocation, particularly given his recent diarrhea, was a possible origin.

When MRI was re-examined following the patient’s seizure, cerebral infarction was initially suspected rather than GBS meningitis or intra-cranial abscess. However, CNS infections account for approximately 15 % of new-onset seizure etiologies in adults, excluding epilepsy [10]. When patients with underlying conditions, such as GBS bacteremia, develop new neurological symptoms, including seizures, GBS meningitis and subdural empyema should be considered in the differential diagnosis.

Although there has been increasing incidence of penicillin resistance, penicillin is still an antibiotic of the first choice for invasive GBS infection. The Infectious Diseases Society of America (IDSA) guideline [11] recommends at least 2 weeks of antibiotic therapy in cases of GBS meningitis. In our case, due to the suspected complication of infective endocarditis (IE), combination therapy of penicillin and an aminoglycoside for its synergistic effects was administered for a duration of 2 weeks. Given progression to subdural empyema in our case, penicillin therapy was extended to eight weeks.

Subdural abscess, a collection of pus between the dura and arachnoid mater, is significantly less common than brain abscess. It typically arises as a complication of purulent meningitis, particularly in pediatric patients and young adults [12]. A review [13] indicates that subdural empyema complicates approximately 2.7 % of cases of adult bacterial meningitis, with 83 % of meningitis cases complicated by subdural abscess showing positive blood cultures. In this case, the same bacterial species were identified in cultures from blood, CSF, and abscess samples. Notably, the initial head MRI revealed no evidence of CNS infection. We hypothesize that the infection progressed sequentially, beginning with bacteremia, advancing to meningitis, and subsequently leading to the formation of a subdural abscess.

The most common causative microorganisms of intracranial subdural abscess include anaerobes, aerobic streptococci (such as GBS), *Haemophilus influenzae*, and other gram-negative bacilli. Diagnosing subdural abscesses can be challenging due to their nonspecific symptoms. Early diagnosis and treatment are critical, as they have a significant impact on patient prognosis [14], [15]. Understanding the disease's natural history and clinical features is therefore essential. The most common presenting symptoms include fever, headache, and vomiting, but focal neurological deficits and seizures may develop as the disease progresses [14], [15]. According to a previous study [13], a substantial proportion of meningitis patients with subdural abscesses experienced complications, such as seizures, respiratory failure, and hemiparesis, within eight hours following CSF sampling. It is hypothesized that this may be due to brain shifts triggered by CSF sampling, even in the absence of imaging evidence of brain herniation. In our case, seizures occurred four hours after the CSF examination, underscoring the importance of closely monitoring patients suspected of having subdural abscesses following such procedures.

Treatment of subdural empyema involves both antimicrobial therapy and surgical drainage to achieve cerebral decompression and infection control [16]. There is debate about the optimal surgical technique for abscess removal. Some literature suggests that craniotomy has better outcomes than burr hole surgery [17], while other studies indicate the surgical method has little impact on the clinical prognosis if surgery is performed promptly and the abscess can be completely removed [18]. Subdural abscesses reaccumulate after drainage in approximately 50 % of cases [19], often requiring repeat drainage. This highlights the need for repeated assessment and, potentially, multiple interventions to manage subdural abscesses effectively. The optimal duration of antibiotic therapy has not established in randomized, controlled study, however, the usual duration of antimicrobial therapy is six to eight weeks [20].

In conclusion, meningitis and subdural empyema should be included in the differential diagnosis for patients with GBS bacteremia who experience neurological deterioration or new-onset seizures. Intracranial complications, such as subdural empyema, can arise during the course of meningitis. Repeat brain imaging is warranted for patients with worsening neurological symptoms during meningitis treatment, even if initial MRI findings are unremarkable.

## Ethical approval

The study is a case report, only information from the patient’s file was used, no type of intervention was performed with the patient, so it does not have approval from the ethics committee.

## Consent

Written consent was obtained from the patient.

## Funding

None.

## CRediT authorship contribution statement

**Jo Onaka:** Writing – original draft. **Takahiro Fukushima:** Writing – review & editing. **Akihito Yoshida:** Writing – review & editing. **Nicole Leedy:** Writing – review & editing. **Takaaki Kobayashi:** Writing – review & editing. **Kyoichi Tomoto:** Writing – review & editing. **Kazuaki Aoki:** Writing – review & editing.

## Declaration of Competing Interest

The authors declare that they have no known competing financial interests or personal relationships that could have appeared to influence the work reported in this paper.
